# Adherence to Antibiotic Prescriptions Among Dental Patients in Saudi Arabia: A Cross-Sectional Study

**DOI:** 10.7759/cureus.78599

**Published:** 2025-02-06

**Authors:** Hamdan Alamri, Turki Almuraikhi, Waleed Alhawiti, Ghaida Alhamad, Lolo Almulla, Norah Almoqhim, Reem Alhurayyis, Rand Alsalamah, Alhussain Daghriri

**Affiliations:** 1 Department of Community Dentistry and Oral Epidemiology, College of Dentistry, Qassim University, Buraydah, SAU; 2 Department of Restorative Dentistry and Prosthodontics, College of Dentistry, Majmaah University, Al Majma'ah, SAU; 3 Department of Dentistry, University Medical Center, Majmaah University, Al Majma'ah, SAU; 4 Department of Dentistry, College of Dentistry, Majmaah University, Al Majma'ah, SAU; 5 Department of Prosthodontics, College of Dentistry, Qassim University, Buraydah, SAU

**Keywords:** antibiotic adherence, antibiotic resistance, antibiotics, bacterial resistance, dental patients, patient adherence

## Abstract

Background: Antimicrobial resistance (AMR) is an increasing worldwide health concern, and non-adherence to antibiotic medication is a crucial factor in its growth. The objective of this research was to assess the adherence of dental patients in Saudi Arabia to oral antibiotic therapy, their knowledge of AMR, and their precise antibiotic prescription.

Methodology: From February 2023 to July 2023, a descriptive quantitative study was conducted among 450 outpatient dental patients who had obtained antibiotic prescriptions from dentists in the previous year. The questionnaire included three elements: knowledge of AMR, antibiotic usage behavior, and demographic information.

Results: During the study, it was found that only 3.6% of participants showed complete adherence to antibiotic therapy; 34% showed adherence to antibiotic therapy to a moderate extent, while 62.3% exhibited low adherence. Statistically significant relationships were established between age and income, and patients’ adherence levels, with considerably lower adherence among the younger and the patients with lower income. Despite 72.5% admitting the importance of appropriate use of antibiotics, 68.8% said they had stopped taking the antibiotic medication once they started to feel better, and 35.3% used lower prescribed doses.

Conclusion: The results of the study suggest that the level of adherence to antibiotic prescription among dental patients in Saudi Arabia is low, and this is of great concern to AMR. It is hereby imperative to tackle the elements that lead to nonadherence to maintain the efficacy of antibiotics and protect public health. Healthcare stakeholders can achieve AMR reduction and optimal patient outcomes by implementing focused interventions and public health efforts.

## Introduction

Since the discovery of penicillin, antibiotics have been crucial in treating infections, including in the field of oral and dental care [1,2]. However, global antibiotic consumption has surged, leading to concerns about antimicrobial resistance (AMR) [3]. Dentists, including pediatric dentists, contribute significantly to this consumption, with reports indicating high rates of unnecessary prescriptions. In 2015, it was reported that a staggering 40%-50% of antibiotic prescriptions worldwide were prescribed unnecessarily [4]. Against this backdrop, there has been an increase in AMR worldwide [5]. Public knowledge and awareness of this issue are hereby considered a prerequisite to ensure the appropriate use of antibiotics to help limit the spread of AMR [6].

With the knowledge that nonadherence to antibiotic therapy can result in several issues, including AMR and treatment failures, D'Ambrosio et al. assessed oral antibiotic medication adherence and AMR awareness among consecutively enrolled dental patients [7]. Before this, there had been relatively little research on AMR awareness among this population. The authors found that dental patients showed a low adherence rate (51.82%) to oral antibiotic therapy. However, adherence rates were notably higher among participants with higher education levels, younger age groups, and those not living with a partner. They concluded that understanding antibiotic therapy, especially regarding AMR, plays a crucial role in improving treatment adherence, which is essential for the effectiveness of the medication [7].

The literature on antibiotic prescribing practices in dentistry underscores the challenges of AMR and the need for prudent prescription practices. Studies have revealed a concerning trend of overprescribing and the inappropriate use of antibiotics in dental settings, with a significant proportion of prescriptions deemed unnecessary [8-10]. Factors contributing to this phenomenon include patient and parental pressure, diagnostic uncertainties, appointment constraints, and social dynamics within dental practices [11]. Pediatric dentists are particularly implicated in unwarranted antibiotic use, highlighting the importance of raising awareness and promoting judicious prescribing practices within this specialty. Despite the growing recognition of the threat posed by AMR, gaps persist in dental practitioners' understanding of its implications and the role of prudent antibiotic use in mitigating resistance development.

In previous studies, the limitations regarding participants' ability to share their experience-based reasoning hindered a comprehensive understanding. In this study, we aim to evaluate patient adherence to oral antibiotic therapy in Saudi Arabia by assessing dental patients' knowledge of AMR and the importance of appropriately prescribing antibiotics to facilitate compliance. This research aims to assess the awareness among Saudi Arabian dental patients regarding the implications of nonadherence to antibiotics and its broader impact on public health. By evaluating the outcomes of nonadherence and gauging dental patients' awareness levels concerning antibiotic adherence, we hope to contribute insights for effectively addressing antibiotic nonadherence and improving overall population health.

## Materials and methods

Study design

This descriptive qualitative and quantitative study targeted outpatients who received antibiotic prescriptions from dentists in Saudi Arabia within one year of conducting the survey. The sample size was calculated to be a minimum of 385 using the Cochran formula.

The inclusion criteria were mentally competent outpatients aged 20-60; there were no gender restrictions. Patients with cognitive impairment and patients who did not fall into the age category were excluded from the study.

The questions were adopted from a previous study, whereby a few were modified, and two were added with 0.8 Cronbach's alpha (see the Appendix) [5]. The questionnaire was structured into three sections. The first section collected the respondents’ demographic information, including age, gender, and education level. The second section presented eight yes or no questions, with modifications to enhance clarity and assess various aspects of antibiotic usage. Notably, adjustments were made to the phrasing of questions to address forgetting doses, reducing doses, and understanding the consequences of improper antibiotic use.

Additionally, two new questions were introduced to evaluate the patients’ understanding of antibiotic usage and its health implications. The final section employed a Likert scale to gauge respondents' frequency of antibiotic use behaviors. The questionnaire was distributed online via platforms such as WhatsApp and Twitter. However, ethical approval was obtained before it was distributed. Subsequently, the collected data underwent analysis and interpretation to derive insights into the respondents’ antibiotic usage patterns and awareness. The study lasted from February to July 2023.

Statistical analysis

The research study used descriptive statistics to summarize the data, including counts, proportions (%), and mean values with standard deviations (SDs), as appropriate. To assess the overall adherence level of the study participants, the researchers built a scoring system comprising seven questions. Questionnaire items 2-6 and 8 required a yes or no answer, while item 7 provided a Likert scale answer (never, rarely, sometimes, often, and always). The questionnaire score was computed as follows: one point was assigned to no answers, and no points were assigned to yes answers, except for item 8, where the scores were inverted (no points were assigned to no answers, one point was assigned to yes answers). For item 7, the answers "never" and "rarely" were assigned a value of 1, while the answers "sometimes," "often," and "always" were assigned a value of 0. The total questionnaire score ranged from 0 to 7, where a score of 7 indicated complete adherence, scores of 6 showed medium adherence, and scores <6 indicated low adherence to antibiotic therapy.

Furthermore, to compare the association between demographic factors and antibiotic adherence, Chi-square and Fisher's exact tests were employed. A significance level of less than 0.05 (p < 0.05) was considered statistically significant. All statistical analyses were performed using the Statistical Package for Social Sciences, version 28 (IBM Corp., Armonk, NY).

## Results

The sociodemographic profile of the study participants (n = 385) highlights several key characteristics. Most respondents were women (n = 310, 80.5%), while men constituted a smaller proportion (n = 75, 19.5%). Age distribution showed that nearly half of the participants (n = 182, 47.3%) were aged 23-30 years, followed by 41-50 years (n = 78, 20.3%), 31-40 years (n = 76, 19.7%), and over 50 years (n = 49, 12.7%). Regarding educational attainment, most participants held a bachelor's degree (n = 275, 71.4%), while others had up to high school education (n = 73, 19%), secondary education (n = 14, 3.6%), higher education such as Masters or PhD (n = 21, 5.5%), and only a small group was uneducated (n = 2, 0.5%). Geographically, the majority were from the Central Region (n = 273, 70.9%), with smaller proportions from the Eastern Region (n = 49, 12.7%), Western Region (n = 30, 7.8%), Northern Region (n = 17, 4.4%), and Southern Region (n = 16, 4.2%). Income levels revealed that the largest group earned less than 7,000 (n = 230, 59.7%), followed by 7,000-10,000 (n = 71, 18.4%), 10,001-15,999 (n = 40, 10.4%), 16,000-20,000 (n = 19, 4.9%), and more than 20,000 (n = 25, 6.5%). The statistical analysis using a t-test indicated that p values for the variables were nonsignificant, suggesting no substantial difference in adherence levels across the demographic groups (Table 1).

**Table 1:** The sociodemographic profile of the population under investigation Data are presented as n (%). p and t values were calculated using the t-test to measure the significance of differences across demographic categories

Variables	Frequency	p value	Test statistic (t value)
Gender			
Female	310 (80.5)	0.042	2.03
Male	75 (19.5)		
Age			
23-30 years	182 (47.3)	0.01	2.67
31-40 years	76 (19.7)		
41-50 years	78 (20.3)		
Older than 50 years	49 (12.7)		
Education level			
Bachelor	275 (71.4)	0.078	1.76
Higher education	21 (5.5)		
Up to high school	73 (19)		
Up to secondary	14 (3.6)		
Uneducated	2 (0.5)		
Residential area			
Central region	273 (70.9)	0.06	1.89
Eastern region	49 (12.7)		
Northern region	17 (4.4)		
Southern region	16 (4.2)		
Western region	30 (7.8)		
Income			
Less than 7,000	230 (59.7)	0.032	2.15
7,000-10,000	71 (18.4)		
10,001-15,999	40 (10.4)		
16,000-20,000	19 (4.9)		
More than 20,000	25 (6.5)		

The data in Table 2 provide insights into antibiotic usage patterns among the study participants. All participants (n = 385, 100%) had taken antibiotics prescribed by a dentist during the year. A notable proportion (n = 260, 67.5%) did not forget to take their antibiotics, while 125 participants (32.5%) reported instances of forgetfulness, though this difference was not statistically significant (t = 1.73, p = 0.084). Regarding reasons other than forgetfulness for not taking antibiotics, 200 respondents (51.9%) identified this issue, with "symptoms disappeared" (n = 94, 47%) being the most cited reason, which was statistically significant (t = 2.12, p = 0.035). Interestingly, 265 participants (68.8%) admitted to discontinuing antibiotics prematurely when feeling better, a highly significant result (t = 3.45, p < 0.001). Regarding proper education on antibiotic usage, 279 respondents (72.5%) acknowledged receiving clear instructions, a significant finding (t = 2.80, p = 0.005). However, challenges in adhering to antibiotic regimens, such as forgetting doses during travel (n = 127, 33%) and reducing dosages (n = 136, 35.3%), were reported but not statistically significant. These findings emphasize gaps in adherence and highlight the critical need for better education and intervention strategies to promote antibiotic compliance.

**Table 2:** Antibiotic usage Antibiotic usage among the study participants. Data are represented as n (%). t test is the statistical test used ^*^A p value of <0.05 indicates statistical significance ^**^A p value of <0.001 indicates high statistical significance

Variables	Frequency	t value	p value
1 Have you taken antibiotics prescribed by a dentist during this year?			
Yes	385 (100)	-	
2. During the specified period for using the antibiotic prescribed by the dentist, did you forget to take it at any time?			
No	260 (67.5)	1.73	0.084
Yes	125 (32.5)		
3. Sometimes people may not take antibiotics, but not because they forget. Were there any days when you did not take antibiotics for reasons other than forgetfulness?			
No	185 (48.1)	2.12	0.035^*^
Yes	200 (51.9)		
If yes, why did not you take antibiotics on those days?			
Fear of side effects of medication	42 (21)	2.01	0.048^*^
No clear instructions about the importance of completing the entire course	64 (32)		
Symptoms disappeared	94 (47)		
4. Have you ever reduced the dosage of the antibiotic and taken fewer doses in a day than prescribed?			
No	249 (64.7)	0.85	0.398
Yes	136 (35.3)		
5. Do you sometimes forget to take antibiotics with you when leaving home or traveling?			
No	258 (67)	0.67	0.502
Yes	127 (33)		
6. When you felt better, did you sometimes stop taking the antibiotics?			
No	120 (31.2)	3.45	<0.001^**^
Yes	265 (68.8)		
7. How often have you faced difficulty in taking antibiotics?			
Always	33 (8.6)	0.98	0.329
Often	51 (13.2)		
Sometimes	40 (10.4)		
Rarely	192 (49.9)		
Never	69 (17.9)		
8. Has the importance of taking antibiotics regularly at the right time and correct dosage, and its impact on overall health, been explained to you?			
No	106 (27.5)	2.8	0.005^**^
Yes	279 (72.5)		

**Figure 1:**
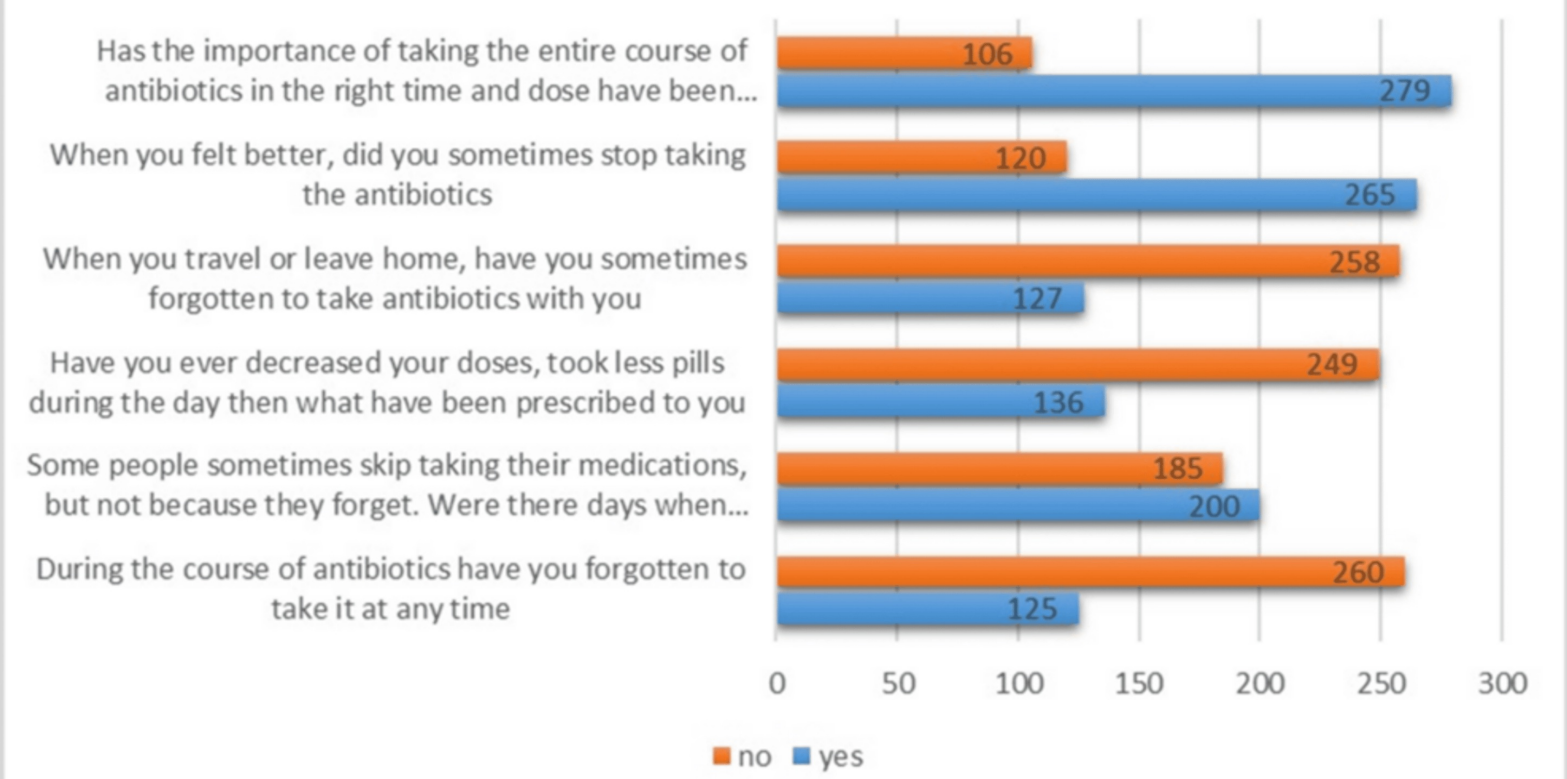
Distribution of the responses to the antibiotic usage questions The Y-axis represents six distinct facets of antibiotic use, while the X-axis shows the number of responses (n = 385). Responses are categorized as "Yes" and "No" and are displayed in blue and orange, respectively

Figure 1 presents the answers to several inquiries about the respondents’ compliance with antibiotic prescriptions. Six questions are listed on the y-axis, each addressing a distinct facet of antibiotic use, while the X-axis shows the number of responses from 0 to 385. The “no” and “yes” responses are indicated on the chart in orange and blue, respectively. The first question inquired whether the respondents had been informed of the significance of taking the antibiotics for the full prescribed duration at the appropriate time and dosage. In this case, 279 respondents (72.5%) indicated that they understood the importance but did not always follow the instructions. Likewise, 265 participants (68.8%) acknowledged that they had stopped taking their antibiotics when they felt better, even though the course had not been finished.

The graph also shows that 127 respondents (33%) neglected to take antibiotics while traveling or leaving home. Furthermore, 136 respondents (35.3%) acknowledged that they had reduced their dosages or taken fewer medications during the day than recommended. Two hundred respondents (51.9%) said that not taking prescriptions for reasons other than forgetfulness was the most prevalent problem. Finally, 125 respondents (32.5%) acknowledged that they had occasionally forgotten to take their antibiotics.

Assessment of the overall adherence level of the study participants

**Figure 2:**
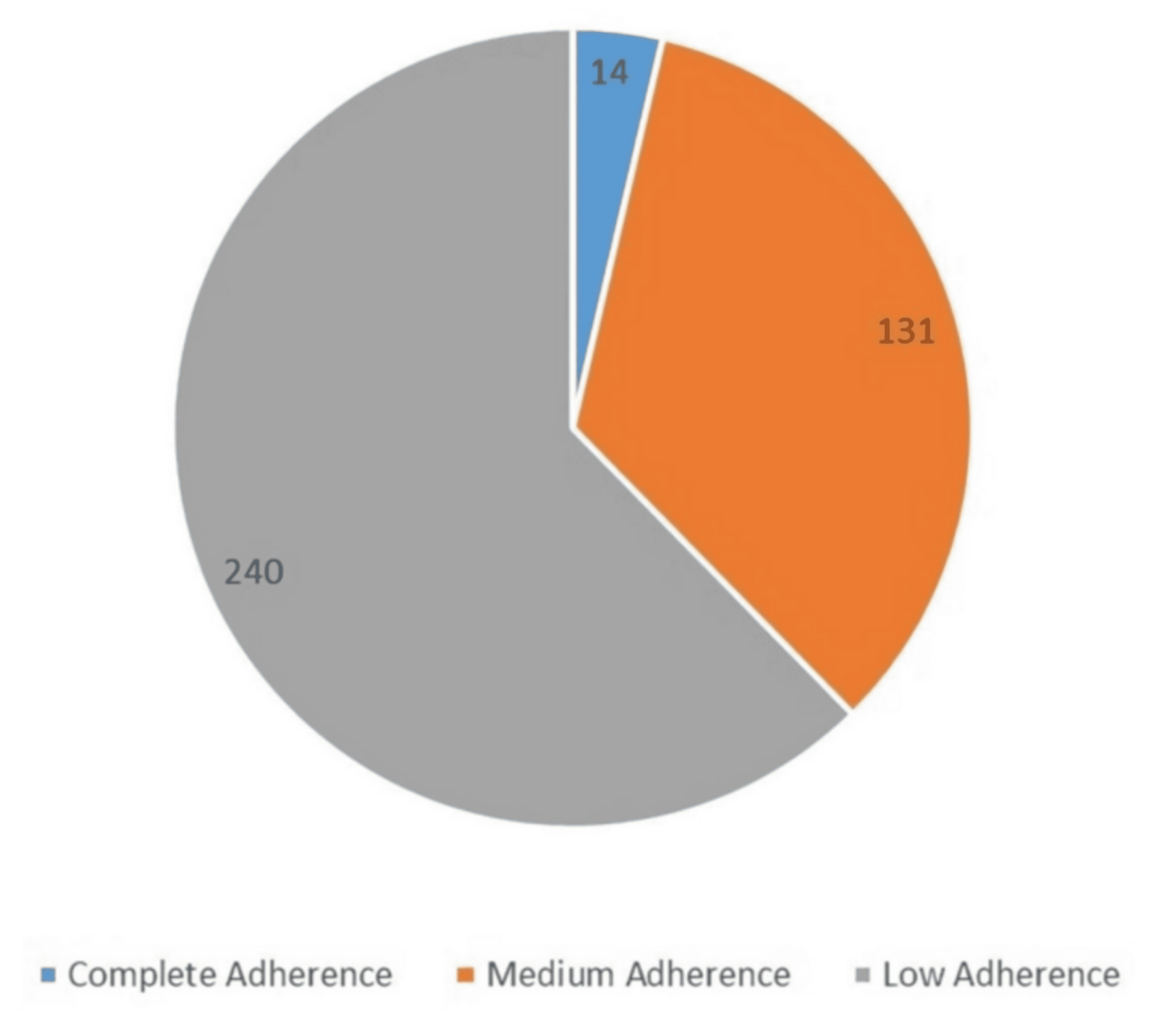
Assessment of the overall adherence level of the study participants Data are represented as the mean adherence score (mean ± SD = 4.18 ± 1.97) with a median of 5. Adherence levels are classified into "complete," "medium," and "low" categories. A p value of <0.05 is considered statistically significant SD: standard deviation

According to the assessment of the overall adherence level of the study participants, only 14 participants (3.6%) demonstrated complete adherence, while 131 (34%) had medium adherence and 240 (62.3%) had low adherence. The mean adherence score was 4.18 out of 8, with a median of 5 and an SD of 1.97 (Figure 2).

Table 3 highlights the distribution of antibiotic adherence levels based on gender, age, education, and income. Among female patients (n = 310, 80.5%), nine (2.9%) demonstrated complete adherence, 101 (32.6%) showed medium adherence, and 200 (64.5%) exhibited low adherence. In comparison, male participants (n = 75, 19.5%) had higher proportions of complete adherence (n = 5, 6.7%) and medium adherence (n = 30, 40%), with fewer participants in the low adherence category (n = 40, 53.3%) (p = 0.091). Age was significantly associated with adherence (p < 0.001), with younger participants (23-30 years, n = 182, 47.3%) exhibiting the highest rate of low adherence (n = 132, 72.5%). Adherence improved with age, with 42.9% (n = 21) of participants over 50 years (n = 49, 12.7%) having low adherence. Education level was not significantly associated with adherence (p = 0.398), though participants with a bachelor’s degree (n = 275, 71.4%) demonstrated the highest medium adherence (n = 94, 34.2%). Income showed a significant association with adherence (p = 0.018), with participants earning less than 7,000 SAR (n = 230, 59.7%) exhibiting the highest low adherence rate (n = 158, 68.7%). In comparison, those earning more than 20,000 SAR (n = 25, 6.5%) showed better adherence, with only 48% (n = 12) in the low adherence category. These results highlight the interplay of demographic and socioeconomic factors on antibiotic adherence levels. The p value was calculated by analysis of variance.

**Table 3:** Association between demographic factors and antibiotic adherence Data are represented as n (%). Statistical significance is assessed using p values, with <0.05 considered significant

Variables	Distribution of adherence levels by gender, age, education, and income			p value
	Complete adherence	Medium adherence	Low adherence	
Gender				
Female	9 (2.9%)	101 (32.6%)	200 (64.5%)	0.091
Male	5 (6.7%)	30 (40%)	40 (53.3%)	
Age				
23-30	6 (3.3%)	44 (24.2%)	132 (72.5%)	<0.001
31-40	1 (1.3%)	28 (36.8%)	47 (61.8%)	
41-50	3 (3.8%)	35 (44.9%)	40 (51.3%)	
Older than 50	4 (8.2%)	24 (49%)	21 (42.9%)	
Education level				
Bachelor	10 (3.6%)	94 (34.2%)	171 (62.2%)	0.398
Higher education (Master, PhD)	0 (0%)	12 (57.1%)	9 (42.9%)	
Uneducated	0 (0%)	0 (0%)	2 (100%)	
Up to high school	3 (4.1%)	22 (30.1%)	48 (65.8%)	
Up to secondary	1 (7.1%)	3 (21.4%)	10 (71.4%)	
Income				
Less than 7,000	9 (3.9%)	63 (27.4%)	158 (68.7%)	0.018
7,000-10,000	1 (1.4%)	27 (38%)	43 (60.6%)	
10,000-15,000	2 (5%)	22 (55%)	16 (40%)	
16,000-20,000	0 (0%)	8 (42.1%)	11 (57.9%)	
More than 20,000	2 (8%)	11 (44%)	12 (48%)	

## Discussion

The study examined dental patients' awareness of antibiotic adherence and the consequences of nonadherence, particularly in the context of AMR in Saudi Arabia. We aimed to determine the effects of nonadherence and the variables affecting patients' attitudes toward antibiotics, emphasizing the impact of sociodemographic variables, including age, gender, income, and education, on adherence rates and the degree of awareness among dental patients about AMR.

The study offers significant new insights into the use of antibiotics among dental patients in Saudi Arabia. With 62.3% of patients classified as having low adherence, 34% having medium adherence, and only 3.6% displaying total adherence, the results show a concerning pattern of low adherence to antibiotic prescriptions. According to the results of previous studies, the level of adherence is reduced over time, as Llor et al. reported a nonadherence rate of 25.2%, which decreased over time in an additional 28.7% of participants [12]. Tong et al. discovered that just 13% of patients followed their antibiotic prescription [13]. However, our results are consistent with the previous studies, D’Ambrosio et al. found that 270 dental patients (51.82%) had low adherence to the recommended oral antibiotic medication, 153 patients (29.37%) had medium adherence, and 98 patients (18.81%) had excellent adherence [7]. Similarly, the study by Menditto et al. found that 59.9% of participants did not comply with their antibiotic prescription [14], and a European study found that 55.8% of participants failed to complete their prescribed antimicrobial therapy [15].

Several factors were found to lower the level of adherence to antibiotic therapy. In our study, the level of adherence was negatively influenced by participant age, with the lowest rate, 72.5%, observed in the 23-30 age group. On the other hand, the results indicated a positive correlation with individuals over the age of 50 who used antibiotics more responsibly. In contrast to our study, Rolnick et al. and Assiry et al. found higher nonadherence rates in younger patients [16,17], whereas Fernandes et al. inconsistently associated non-adherence with increasing age [18].

Income also revealed a significant effect on the level of adherence, which was poorer for the group of patients earning “less than 7,000 SAR”; 68.7% exhibited low adherence. Most of the respondents in the “more than 20,000 SAR” income group had a low level of adherence (48%). In the study by Lee et al., the study result showed that patients with low income have higher excess risk due to nonadherence [19]. However, poor education standards among the respondents, who only had up to secondary education or were illiterate individuals, were associated with poor compliance. The proportion of patients with a bachelor’s degree or more education was also significantly related to good compliance with antibiotic therapy. Assiry et al. and Desta et al. reported a higher compliance rate in highly educated patients [17,20].

Currently, no correlation exists that showcases the significance of antibiotic therapy concerning gender, as per studies conducted in a meta-analysis. On the other hand, Manteuffel et al. have documented that self-reported adherence to antibiotics is lower in the female gender [21]. Our study also shows 64.5% low adherence in female patients. Assiry et al. also reported that male patients had higher antibiotic compliance than female patients (59.4%) [17].

The study's results highlight the crucial need to address dental patients' nonadherence to antibiotics to reduce the risks of AMR. It is critical to develop patient education initiatives, foster better communication during prescription dispensing, and increase public knowledge of the adverse effects of incorrect antibiotic use [22]. Fighting the emergence and spread of AMR can be significantly aided by implementing antimicrobial stewardship programs and infection control strategies [23].

The finding found that 68.8% of the respondents stopped taking the antibiotics when they felt better, which is consistent with a European study that found that 55.8% of patients failed to complete the recommended antimicrobial therapy [16]. The present study's results are consistent with those of Chan et al., indicating that low knowledge of antibiotics, including the problem of AMR, affects patients' adherence to antibiotic treatment [24]. This lack of information may cause patients to disregard antibiotic treatment or even deliberately fail to follow it appropriately, unaware of the potential harm to both their own and public health. Raupach-Rosin et al. found that knowledge and awareness of AMR are positively impacted by higher education, especially a university degree [25]. These results highlight the importance of accurately educating dental patients about antibiotic prescription and AMR, with the primary objective being to improve their comprehension of the advantages of appropriate antibiotic usage and the dangers of abuse and misuse, specifically to enhance their general antibiotic-ingestion behavior.

Appropriate antibiotic therapy is essential to recover from bacterial infections [20]. This necessitates using the optimum antibiotic at the suggested dosage, frequency, and duration. However, for the treatment to be successful, the patient must strictly stick to the antibiotic regimen. The overuse of antibiotics can cause harmful bacteria to become resistant, making it more difficult to treat bacterial infections in the general population [26]. Our study found that healthcare practitioners had a significant impact on patients' compliance with antibiotic therapy. As a result, the public has to be well informed about the risks of failing to adhere to antibiotic therapy.

It is important to recognize several limitations, such as using self-reported data, which could be affected by social desirability and recollection bias. The study's cross-sectional nature makes it more difficult to determine causality, and using an online survey may have added bias in the sample selection process. Furthermore, because the sample primarily consisted of people who had access to the Internet and were ready to take part in online surveys, it may not be entirely representative of the larger population of dental patients in Saudi Arabia. Another drawback of this research is the high participants ratio of women to men due to their greater willingness to participate in health-related surveys, higher healthcare-seeking behavior, or cultural factors influencing accessibility and response rates.

Notwithstanding these drawbacks, the research offers insightful information about Saudi Arabian dentistry patients' understanding of AMR and antibiotic adherence. An extensive questionnaire covering demographic traits, antibiotic usage behavior, and knowledge of AMR increases the robustness of the results. The statistical analysis and large sample size further enhance the dependability of the results.

## Conclusions

In summary, the study offers insightful information about Saudi Arabian dentistry patients' awareness of and behavior related to antibiotic adherence. This study's findings emphasize the necessity of stepping up initiatives to encourage Saudi Arabian dentistry patients to adhere to antibiotic treatment regimens and be aware of AMR risks. To minimize the risks associated with AMR and ensure the best possible patient outcomes, it is imperative to address the factors contributing to nonadherence. Healthcare stakeholders can contribute to maintaining public health and protecting the efficacy of antibiotics by implementing focused interventions and public health programs.

## Appendices

**Table 4:** Questionnaire

Demographic data	Options (English)	Options (Arabic)
Gender	Male, Female	ذكر، انثى
Age	23-30, 31-40, 41-50, older than 50	23-30، 31-40، 41-50، 50 أكثر من
Education level	Not educated, Up to secondary, Up to high school, Bachelor, Higher education (Master, PhD)	ابتدائي، متوسط، ثانوي، جامعي، دراسات عليا (ماجستير، دكتوراه)
Range of income	Less than 4,000, 4,000-10,000, 10,000-20,000, more than 20,000	4,000 أقل من، 4,000-10,000، 10,000-20,000، 20,000 أكثر من
Study questions		
1. Have you taken oral antibiotics by a dentist within a year?	A. Yes, B. No	.2 نعم، .1 لا
2. During the course of antibiotics, have you forgotten to take them at any time?	A. Yes, B. No	.2 نعم، .1 لا
3. Were there days when you didn't take your antibiotics? (If yes, why?)	A. Yes, B. No	.2 نعم، .1 لا
4. Have you ever decreased your doses or took fewer pills than prescribed?	A. Yes, B. No	.2 نعم، .1 لا
5. When you travel or leave home, have you sometimes forgotten to take antibiotics with you?	A. Yes, B. No	.2 نعم، .1 لا
6. When you felt better, did you sometimes stop taking the antibiotics?	A. Yes, B. No	.2 نعم، .1 لا
7. How often did you have difficulty remembering to take antibiotics?	A. Never, B. Rarely, C. Sometimes, D. Often, E. Always	.3 مطلقًا، .2 نادرًا، 1. اغلب الأحيان، .5 غالبًا، .4 دائمًا
8. Has the importance of taking the entire course of antibiotics at the right time and dose been explained during the prescription?	A. Yes, B. No	.2 نعم، .1 لا
